# Random walk hierarchy measure: What is more hierarchical, a chain, a tree or a star?

**DOI:** 10.1038/srep17994

**Published:** 2015-12-10

**Authors:** Dániel Czégel, Gergely Palla

**Affiliations:** 1 Dept. of Biological Physics, Eötvös University, H-1117 Budapest, Hungary; 2 MTA-ELTE Statistical and Biological Physics Research Group, Hungarian Academy of Sciences, H-1117 Budapest, Hungary; 3 Regional Knowledge Centre, Eötvös University, H-8000 Székesfehérvár, Hungary

## Abstract

Signs of hierarchy are prevalent in a wide range of systems in nature and society. One of the key problems is quantifying the importance of hierarchical organisation in the structure of the network representing the interactions or connections between the fundamental units of the studied system. Although a number of notable methods are already available, their vast majority is treating all directed acyclic graphs as already maximally hierarchical. Here we propose a hierarchy measure based on random walks on the network. The novelty of our approach is that directed trees corresponding to multi level pyramidal structures obtain higher hierarchy scores compared to directed chains and directed stars. Furthermore, in the thermodynamic limit the hierarchy measure of regular trees is converging to a well defined limit depending only on the branching number. When applied to real networks, our method is computationally very effective, as the result can be evaluated with arbitrary precision by subsequent multiplications of the transition matrix describing the random walk process. In addition, the tests on real world networks provided very intuitive results, e.g., the trophic levels obtained from our approach on a food web were highly consistent with former results from ecology.

Hierarchical organisation is an ubiquitous feature of a large variety of systems studied in natural- and social sciences. Examples of empirical studies on hierarchy are including the transcriptional regulatory network of Escherichia coli^1^, the dominant-subordinate hierarchy among crayfish^2^, the leader-follower network of pigeon flocks^3^,^4^, the rhesus macaque kingdoms^5^, neural networks^6^ and technological networks^7^, scientific journals^8^, social interactions^9^,^10^,^11^, urban planning^12^,^13^, ecological systems^14^,^15^, and evolution^16^,^17^. Naturally, hierarchy is a very relevant concept also in network theory^7^,^18^,^19^,^20^. The network approach has become an ubiquitous tool for analysing complex systems ranging from the interactions within cells through transportation systems, the Internet and other technological networks to economic networks, collaboration networks and the society^21^,^22^.

Grasping the signs of hierarchy in networks is a non-trivial task with a number of possible different approaches. On the one hand, we may try the statistical inference of an underlying hierarchy based on the observed network structure, as suggested in ref. 19. On the other hand, the introduction of a hierarchy measure is also a natural idea^23^,^24^,^25^,^26^,^27^. In general, a hierarchy measure, can be viewed as a function on the domain of graphs, 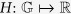, mapping a graph 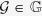 into a real number, 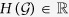. The value of the measure is actually 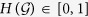 or 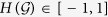 in most cases, with high values corresponding to hierarchical structures and low values indicating the absence of hierarchy in the examined network.

One of the first methods was proposed by D. Krackhardt, motivated by organisational hierarchy, defining the hierarchy measure simply as the number of ordered pairs divided by the number of connected pairs^23^. In the approach introduced by A. Trusina *et al*., the position of the nodes in the hierarchy is assumed to be given by the degree, and the hierarchy measure is given by the fraction of directed shortest paths going strictly upwards in the hierarchy^24^. Another way for quantifying the possible asymmetry between nodes is to assume some sort of flow on the links, and examine whether the global map of flows in the system is revealing a kind of overall directionality or not. Probably the simplest approach in this framework is to define the fraction of links not participating in any cycle as the measure of the hierarchy, as suggested by J. Luo and C. L. Magee^25^.

A further important property of a hierarchical system is that reaching the rest of the network should be relatively easy for the nodes high in the hierarchy, and more difficult for the nodes at the bottom of the hierarchy, as pointed out by E. Mones *et al*. in ref. 26. The hierarchy measure based on this aspect is given by the Global Reaching Centrality, characterising the inhomogeneity of the fraction of reachable nodes in at most *m*-steps in the network^26^. A more elaborate quantification of hierarchy was proposed by B. Corominas-Murta *et al*. in ref. 27 with the introduction of Treeness, Feedforwardness and Orderability, projecting the studied network onto a point in a 3 dimensional space, where each dimension is aimed to capture a different aspect of hierarchy. Treeness, *T*, is measuring how ambiguous are the chain of commands in the network, while Feedforwardness, *F* is related to the size and position of the strongly connected components in the network. Finally, the orderability, *O* is simply the fraction of nodes not taking part in any directed cycles, i.e., it is analogous to the hierarchy measure introduced by J. Luo and C. L. Magee. The 3d scatter plots of *T*, *F* and *O* provided very interesting results, revealing different clusters of hierarchical networks^27^. A more detailed description and comparison between the mentioned methods is given in the Supplementary Information S1.

Although the methods listed above allow the examination of the hierarchical organisation from different perspectives, a noteworthy common aspect of these approaches that they all treat acyclic networks as already maximally hierarchical, independent of the further details of the graph structure. (The Global Reaching Centrality given in ref. 26 is an exception, considering the star configuration as the most hierarchical). Here we argue that different acyclic networks are not necessarily equally hierarchical. The general intuition of a hierarchy is usually corresponding to a multi level pyramidal structure, with levels becoming wider and wider as we descend from the root towards the bottom. On the one hand this way the top nodes in the hierarchy can reach most of the network in a very effective way, i.e., via paths of average length scaling as ln *N*, where *N* denotes the number of nodes. On the other hand, in this structure all nodes can have a treatable number of direct subordinates. In contrast, if we consider a directed chain, all the levels are of size one, and this is leading to a large average distance scaling as *N*. The other extreme limiting case of acyclic networks is given by the directed star configuration, where all the nodes have a single incoming link from a central hub, and no further out-links. In this case the hierarchy is consisting of only two levels, and the supposed leader in the network has to cope with a number of direct descendants scaling as *N*. Based on that, introducing a hierarchy measure preferring trees to chains and stars would be a substantial step towards achieving a more intuitive approach for evaluating the importance of hierarchy in a network structure.

In this paper we tackle this problem with the help of random walks on the network. Random walks provide a fundamental model for stochastic processes in a large variety of systems ranging from physics^28^, chemistry^29^ and computer science^30^ through biology and ecology^31^,^32^ to economics^33^ and psychology^34^. In the current problem of quantifying the extent of hierarchy in a network structure, random walkers can be used to evaluate the rank of the nodes in the hierarchy.

The basic idea is assuming an information flow on the links from nodes high in the hierarchy towards the lower levels, in a similar fashion as in case of a company, where the management is likely to send information and instructions to the employees on a regular basis. Given the network structure, the source of information in the system can be traced back by sending random walkers traversing the links in reverse direction from all nodes. In case the density of the random walkers is reaching a steady state, its value at a given node can be interpreted as the probability that the node was the source of information. Consequently, high random walker density values indicate a high standing in the hierarchy, whereas low density values are corresponding to bottom nodes. The significance of hierarchical organisation in the network structure can be judged based on the inhomogeneity of the distribution: In a homogeneous distribution we cannot pinpoint the source of information, thus, it is corresponding to a non-hierarchical network. In contrast, a very inhomogeneous distribution is indicating a strongly hierarchical structure.

## Random walk hierarchy measure

The details of the random walk process are the following. Since the random walkers are traversing the links backwards, the transition probability for a walker from node *j* to *i* is proportional to the inverse of the in-degree of *j*, i.e., 
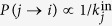
. Another important factor to be taken into account is the limited capacity of the information sources for sending information: In general we can assume that the more out-neighbours a given node has, the less resource it can allocate for managing the communication over a given link. This effect can be taken into account by assuming that *P*(*j* → *i*) is also proportional to the out-degree of *i*, i.e., 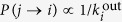. Combining the above factors together is resulting in


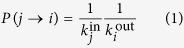


for the transition probability of the random walkers from node *j* to *i*. (In case *i* is not an in-neighbour of *j* the transition probability *P*(*j* → *i*) is zero by definition). We note that due to the second factor on the right hand side of (1), the probability for staying at the same node can be non-zero in general, given by 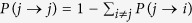. For weighted networks (1) can be naturally generalised to


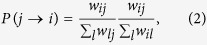


where *w*_*ij*_ denotes the weight of the link from *i* to *j*.

In case of acyclic networks, all random walkers eventually converge into nodes with no incoming links, (i.e., the ‘sources’ in the network). In order to avoid judging the importance of hierarchical organisation in the system solely based on these ‘sources’, we inject new random walkers into the network at every time step. The update rules are the following:

1. We insert *f* random walkers into the system, increasing the random walker density on every node by *f*/*N*, thus, the random walker density at node *i* given by *p*_*i*_(*t*) is changing as


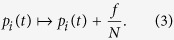


2. We let all random walkers in the system proceed on step, governed by the transition probabilities given in (1). By introducing a transition matrix **T** with matrix elements *T*_*ij*_ = *P*(*j* → *i*), the density of random walkers on node *i* after the transition can be expressed as


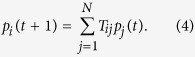


3. The total sum of random walkers has to be normalised, i.e., we require 
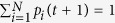
. Since the sum of new random walkers added to the system was *f*, we have to simply divide the density of random walkers by 1 + *f* in order to fulfil the normalisation condition,


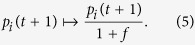


The above normalisation of the random walker density to unity after each iteration is equivalent to using ‘decaying’ random walkers, having a weight decreasing by a factor of (1 + *f*)^−1^ in each step. Let us denote the characteristic distance under which the weight of a random walker is decreased to *e*^−1^ by *λ*, fulfilling


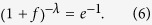


Based on that, *f* can be also expressed as


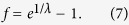


Although *λ*, (or equivalently, *f*) is a parameter of the method at the current stage, later on in the Results we shall find a natural condition for fixing *λ* at an optimal value in general.

Our main object of interest is the stationary distribution of the random walkers in the network. By writing this distribution in a vector form of **p**^stat^, where the *i*-th component of the vector, 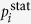, is corresponding to the random walker density on node *i*, we can derive a simple equation based on the update rules. Adding *f*/*N* new random walkers at each node is simply incrementing each vector component by *f*/*N*, while the transition to the next site by the random walkers corresponds to multiplying by the transition matrix **T**. Finally, the normalisation of the random walker density simply multiplies each vector component by 1/(1 + *f*). Based on the above the stationary distribution fulfils


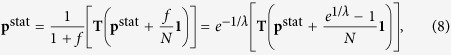


where **1** is corresponding to a vector of size *N* with all elements equal to 1. By expressing **p**^stat^ we obtain


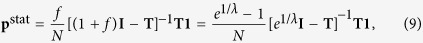


where **I** is denoting the identity matrix. Since **T** is a left stochastic matrix, the absolute value of its largest eigenvalue is 1. Consequently, the absolute value of the eigenvalues of  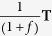  are smaller than 1, and therefore, (9) can also be written as


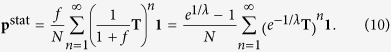


This formula is very intuitive, showing explicitly that the stationary distribution of random walkers at a given node is given by the sum of the probabilities of all the paths ending on the node, where the contributions from longer paths are suppressed exponentially as a function of the path length. Based on (9–10), **p**^stat^ can be computed very efficiently. If the size of the network is moderate, we can use (9) for obtaining exact results. However, if matrix inversion is becoming computationally expensive, a very good approximation of **p**^stat^ can be calculated according to (10). I.e., by carrying out the summation up to a certain finite limit *n*_max_, the obtained result is converging to the exact **p**^stat^ exponentially fast.

Since **p**^stat^ is describing the stationary distribution of random walkers in the network, it is naturally related to PageRank^35^. In Sect.S6. in the Supplementary Information we examine the correlation between 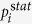 and the PageRank of the nodes in a couple of real systems. According to the results, these two quantities show moderate positive correlations on average, with quite large variations in the correlation coefficients from system to system. (In other words, the correlation is quite strong in some of the studied networks, and almost zero in others). A plausible explanation for this behaviour is that in our model the transition probability depends on the node degrees at both ends of a given link, which can result in substantial differences in the stationary distributions compared to traditional random walk dynamics. Therefore, by evaluating **p**^stat^ we do not ‘reinvent’ PageRank, instead we measure a quantity that can behave quite distinctly compared to PageRank.

Our hierarchy measure is based on the inhomogeneity of the stationary distribution of the random walkers. There are several different possibilities for quantifying the inhomogeneity of a probability distribution in general, here we choose the relative standard deviation, (also called as the coefficient of variation). Thus, the random walk hierarchy measure is defined as


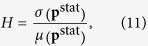


where *μ*(**p**^stat^) and *σ*(**p**^stat^) denote the mean and the standard deviation of 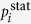 respectively. Since 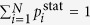, the mean is given simply by *μ*(**p**^stat^) = 1/*N*, and our hierarchy measure can be expressed as


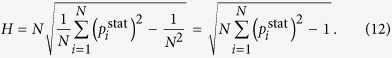


Choosing the coefficient of variation for characterising the inhomogeneity of the stationary distribution of the random walkers has several advantages. First, it is invariant under linear transformation of **p**^stat^, thus, its value is not affected if for example, the stationary distribution of the random walkers is not normalised. Moreover, the 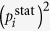 factor appearing in (12) has a very intuitive meaning, since it can be interpreted as the probability to find node *i* as the source of information in two independent ‘experiments’ of sending random walkers traversing the links backwards according to our dynamics. In a strongly hierarchical network the sources of the information are less ambiguous compared to a non-hierarchical network, and accordingly, the probability for finding the same sources in two independent ‘experiments’ is higher.

In addition, a further very important advantage of (11–12) is that according to the results detailed in the section Hierarchy of acyclic networks, *H* is converging to a finite non-zero value for infinitely large regular trees on the one hand, and to zero in case of chains and stars on the other hand. In Sect.S2. in the Supplementary Information we show that this property is lost if we switch from the coefficient of variation to the average difference between 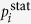 and the maximum of 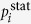, a choice similar to the definition of the Global Reaching Centrality introduced by E. Mones *et al*.^26^. According to the results, the hierarchy measure obtained in this way is still preferring trees over chains or stars, but in this case the value of the measure is converging to zero in the thermodynamic limit for all regular acyclic graphs. (Only the magnitude of the decay exponent is smaller for trees compared to trees and stars). Based on the above, measuring the inhomogeneity of 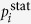 by using the coefficient of variation seems to be an optimal choice for our purposes.

## Results

### Hierarchy of acyclic networks

For demonstrating the sensitivity of our measure to the topology also in case of acyclic networks, first we evaluate *H* for chains, regular trees with a constant branching number *b*, and stars. According to calculations detailed in Methods, the corresponding hierarchy values can be expressed as


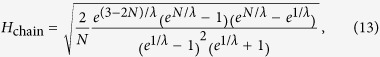



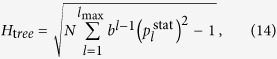



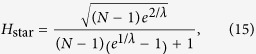


where *N* is the number of nodes in the networks, *l* denotes the levels in case of the tree (starting from *l* = 1 at the root and ending with *l*_max_ at the leafs), and 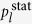 is corresponding to the stationary distribution of the walkers on level *l* in (14), which can be obtained from a simple recursion written as


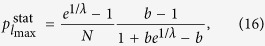



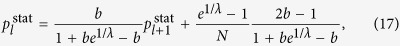



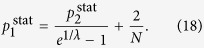


**Figure 1:**
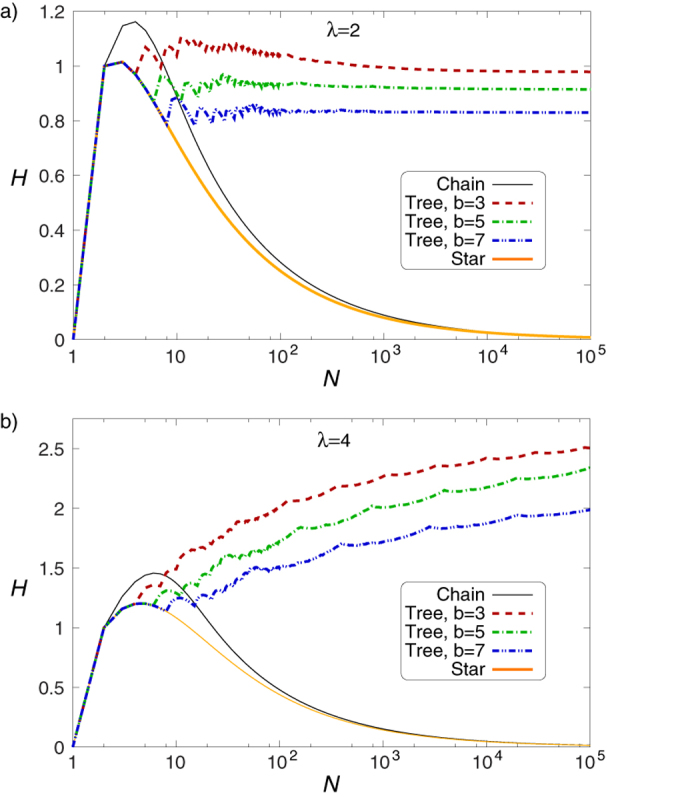
Comparing the random walk hierarchy for chains, regular trees and stars. (**a**) The behaviour of *H* as a function of *N* for a chain (black), a regular tree with branching number *b* = 3 (red), a regular tree with *b* = 5 (green), a regular tree with *b* = 7 (blue) and a star (orange) at *λ* = 2. (**b**) The same plot as in (**a**) when *λ* is set to *λ* = 4.

In Fig. 1 we compare the hierarchy measures given in (13–15) at *λ* = 2 (Fig. 1a) and at *λ* = 4 (Fig. 1b). Our construction algorithm for the trees with a branching number *b* was to start by adding *b* links to the root, then move to the second level and subsequently add *b* links to every node on this level, and so on, move to the next level only when the given level was completed. Whenever the number of nodes in the tree has reached *N*, the algorithm terminates, and naturally, the resulting tree is not completely regular in most of the cases. Nevertheless, the overall structure of the trees obtained in this way is getting closer and closer to regular trees as *N* is increasing.

According to Fig. 1 the *H* for the chain and the star configurations has a peak at very small system sizes, and shows a decreasing tendency for growing *N*. In contrast, for regular trees *H* seems more or less converging to a finite value. Thus, we have achieved our primary goal, the obtained hierarchy measure is preferring trees over chains or stars. For comparison, in Sect.S7.1. in the Supplementary Information we examine the behaviour of the previously introduced alternative one dimensional hierarchy measures from this respect. The results indicate that none of the other hierarchy measures in the study has this property, since they either treat all acyclic graphs as already maximally hierarchical, or assign the largest hierarchy score to stars instead of regular trees.

The results shown in Fig. 1 also suggest that above a certain *N* it is the structure of the tree, (encoded in the branching number), what determines the hierarchy measure, not the size of the tree. This is indicating that *H* is behaving similarly to intensive quantities in physics in some aspects. The ‘intensive’ property of the hierarchy measure is analysed in more details in the Supplementary Information S3, here we note that if we take a pair of graphs 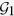 and 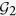 which are not connected to each other, then *H* for the union of the graphs is equal to the weighted quadratic mean of the *H* values calculated for the graphs separately,


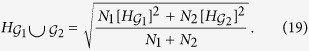


Thus, in the special case of a pair of isomorphic graphs 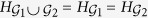.

We continue with the examination of the behaviour of *H* in the thermodynamic limit. According to calculations detailed in Methods, when the system size is diverging, *N* → ∞, the hierarchies given in (13–15) take the form of


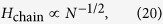



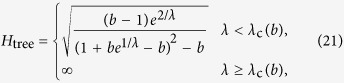



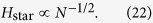


Thus, the hierarchy measure is vanishing for a chain and a star in the thermodynamic limit. In contrast, *H*_tree_ is converging to a well defined finite limit value or *b* < 1 < ∞ when *λ* is smaller than a *b* dependent critical value, and is diverging otherwise. In the Methods we show that the critical *λ* value is given by


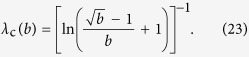


**Figure 2:**
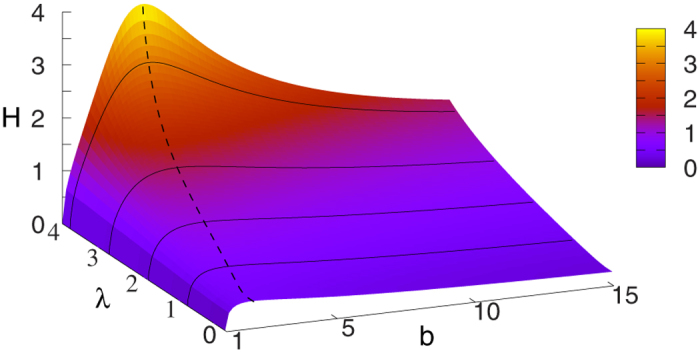
2d plot of the random walk hierarchy measure *H* for infinitely large regular trees as a function of the branching number *b* and the parameter *λ*. The formula for *H* is given in (21). At *b* = 1 we recover the infinitely large chain, while the infinitely large star is corresponding to the *b* → ∞ limit. The dashed line is showing the maximum place of *H*.

The behaviour of the limiting *H*_tree_ given in (21) is shown in a 2d plot in Fig. 2 as a function of *b* and *λ*. At *b* = 1 the tree becomes equivalent to an infinitely large chain, and according to (20) the *H* becomes zero. The 2d surface displayed in Fig. 2 is consistent with this result, as it starts from *H* = 0 at *b* = 1 for all *λ* values. Similarly, the *H* for an infinitely large star is also zero according to (22). The surface shown in Fig. 2 is consistent with this result as well, as we can see a decreasing tendency in *H* as a function of *b* in the large *b* regime. In the range of intermediate branching numbers we can observe an *λ* dependent maximum in *H*. This behaviour is examined in more details in the Supplementary Information S4.

Based on the behaviour of *H* in the thermodynamic limit, we can also fix the *λ* parameter at an optimal value in general as follows. Since *λ* is corresponding to the characteristic path length a random walker can traverse before ‘decaying’, on the one hand we would like to choose a *λ* as high as possible. I.e., if *λ* is small, the random walkers can explore only within a very limited range from their origin, thus, the information we can retrieve via the random walkers is also very local. However, due to its self similar nature, hierarchical organisation can manifest on all length scales, therefore, we need random walkers travelling longer distances in order to be able to tell apart hierarchical and non-hierarchical networks.

On the other hand, if *λ* is too large, we may run into diverging hierarchy values according to (21), which needs to be avoided in case of a well behaving hierarchy measure. Therefore, we fix *λ* at a value as high as possible where a diverging *H* is avoided for sure even in case of infinitely large regular trees. According to (23), the minimum of *λ*_c_(*b*) can be found at *b* = 4, where 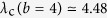. Since the path length traversed by a random walker is increasing by unity under every iteration, it is also natural to set *λ* to an integer value. Based on the above, the optimal setting for *λ* is given by *λ* = 4. In the rest of the paper we are assuming that *λ* is set to this optimal value, and thereby consider our approach a parameter free method for measuring the amount of hierarchical organisation in the structure of networks.

**Figure 3:**
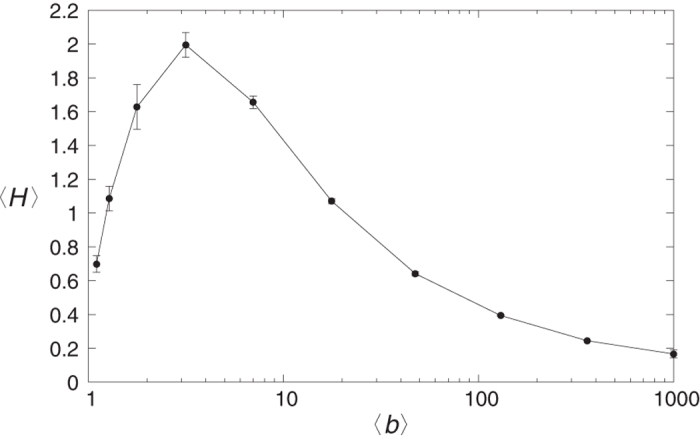
The average random walk hierarchy 〈*H*〉 as a function of the average branching number 〈*b*〉 for general trees of *N* = 1000 nodes, averaged over 100 instances. When 〈*b*〉 is close to one, the tree is basically a chain, whereas at very large branching number, its structure is close to a star.

Since real world hierarchies are usually not as highly ordered as a regular tree with a constant branching number, we extended our comparison studies of acyclic graphs also to general directed trees. By applying a simple algorithm detailed in Methods, we generated a large family of trees with branching numbers varying around a given average branching number 〈*b*〉 according to a shifted Poisson distribution. In Fig. 3 we show the average of the random walk hierarchy measure, 〈*H*〉 as a function of 〈*b*〉, calculated based on 100 realisations of trees consisting of *N* = 1000 nodes. According to the curve, the maximum of 〈*H*〉 is at an intermediate average branching number, where the structure of the network is really tree like. I.e., for low average branching numbers, (where the structure is basically a chain), and also for very large branching numbers comparable to the system size, (where the structure is close to a star), the obtained 〈*H*〉 values are considerably lower.

### Results on real networks

#### St. Marks food web

Here we apply our method for analysing the hierarchy of the St. Marks food web^36^, representing a part of the ecosystem of Goose Creek Bay, St. Marks National Wildlife Refuge, Florida, USA. The nodes of the network are corresponding to living compartments, (group of species) based on probable diet and life history characteristics. Thus, compartments range from single species (e.g., pinfish) through a couple of species (e.g., gulf flounder and needlefish) to large groups of taxa, (e.g., bacterioplankton). The links between the nodes represent the feeding pathways, pointing from consumers to their food sources, where the link weights are corresponding to the fractions of the consumer’s diet.

The static distribution of the random walkers on the network defined above can be calculated using (9). However, 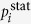 is defining only a ranking between the nodes and does not provide the hierarchy levels in the first place. Therefore, we sampled and aggregated nodes into levels so that in each level, the standard deviation of 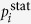 is lower than a pre-defined fraction of the standard deviation in the whole network. (This type of procedure for obtaining the hierarchy levels was established in^26^).

**Figure 4:**
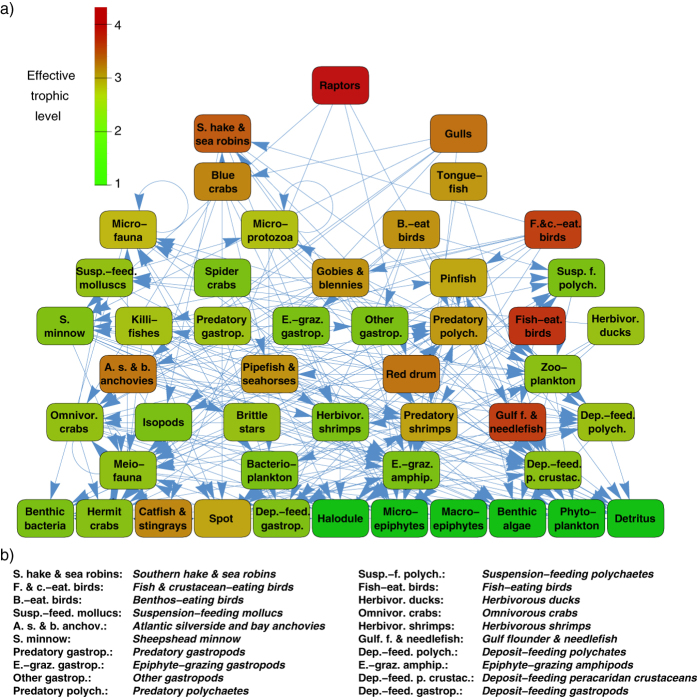
Hierarchy of the St. Marks food web. (**a**) The nodes are ordered according to the stationary distribution of the random walkers calculated from (9), and the hierarchy levels are corresponding to groups of nodes for which the standard deviation of 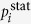 is at most 0.125 ⋅ *σ*(**p**^stat^), where *σ*(**p**^stat^) denotes the standard deviation of 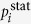 over the whole network. The colour coding of the nodes reflects their effective trophic level published in^36^. (**b**) Listing of the abbreviations used in (**a**).

In Fig. 4 we show the resulting hierarchy between the compartments when the standard deviation of 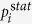 within the levels is at most 0.125 ⋅ *σ*(**p**^stat^). The hierarchy levels are consistent with the common sense about food webs as e.g., benthic algae is on the lowest level, herbivorous ducks are somewhere in the middle, and raptors (such as e.g., the bald eagle) are on the top of the hierarchy. The colour coding of the nodes is showing the effective trophic level of the compartments given in^36^, ranging between 1.0 and 4.32. Apparently, the position of the nodes in the hierarchy and their colour are coherent in most of the cases, e.g., the root has the highest effective trophic level, and the nodes with the lowest trophic level are at the bottom of the hierarchy. However, a small number of discrepancies can be also observed, (e.g., as in case of Gulf flounder & needlefish), signing that the effective trophic levels and the random walk based hierarchy are catching slightly different aspects of the studied food web.

Finally, the Spearman’s rank correlation coefficient between the ranking of the compartments according to 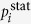 and the ranking according to the effective trophic levels is 0.593. In contrast, the Spearman’s rank correlation coefficient between the effective trophic levels and the hierarchy obtained after applying a degree preserving link randomisation to the network is only 0.006 ± 0.138. Based on the above, our hierarchy is highly consistent with former results from ecology.

### Comparing different networks

**Figure 5:**
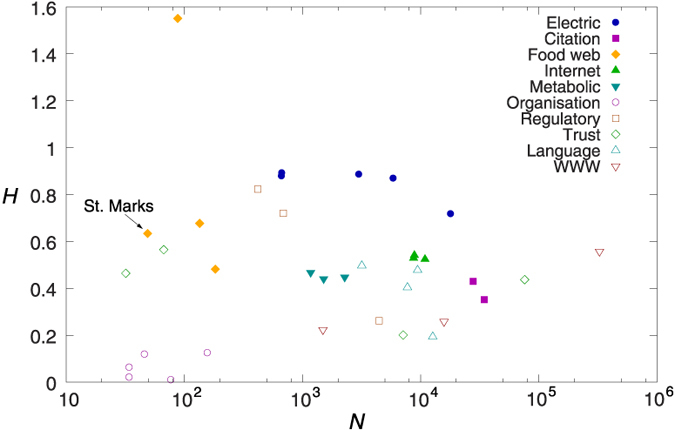
Random walk hierarchy of different real networks. Each symbol is corresponding to a different network, where the shape and colour of the symbols is encoding the type of the system. The horizontal coordinate of the symbols is corresponding to the size of the corresponding network, while the vertical coordinate is giving *H*.

We also calculated the *H* given in (12) for numerous different systems ranging from metabolic and regulatory networks through citation, trust and language networks to the Internet and the WWW. (A detailed description of the networks is given in the Supplementary Information S5). In Fig. 5 we show the obtained hierarchy values as a function of the network size, *N*. According to the figure food webs, electric circuits and regulatory networks provide the largest *H* values, and in contrast, the informal networks of acquaintances in different organisations seem the least hierarchical. In the mean time, the WWW, the Internet, the citation-, metabolic-, trust- and language networks appear to be moderately hierarchical.

However, under certain circumstances we may obtain a moderate hierarchy measure even in a random graph. E.g., the structure of the giant component in the Erdös–Rényi graph^37^ is more or less tree-like if we are close to the percolation threshold, and tree-like structures are usually considered highly hierarchical. Accordingly, in order to make a fair judgement on the importance of hierarchy in the topology of a real network, we need to compare the measured *H* to the result we expect in a suitably chosen random network ensemble, modelling the structure of the given network under the assumption of random connections. In order to take into account of the degree distribution of the studied networks, we use the configuration model for evaluating the expected value of *H* in the random network ensemble. A sample from this ensemble can be obtained by simply link randomising the given real network, keeping the degree of the nodes fixed under the random rewiring of the connections.

The difference between *H* obtained for the real networks and the expected value of *H* in their random counterparts can be measured in terms of the *z*-score, defined as


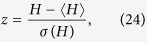


where 〈*H*〉 and *σ*(*H*) denote the expected value- and the standard deviation of *H* in the random ensemble, respectively. Thus, we basically scale the difference between the real *H* and the average of *H* over the random ensemble by the standard deviation of *H* in the random ensemble.

**Figure 6:**
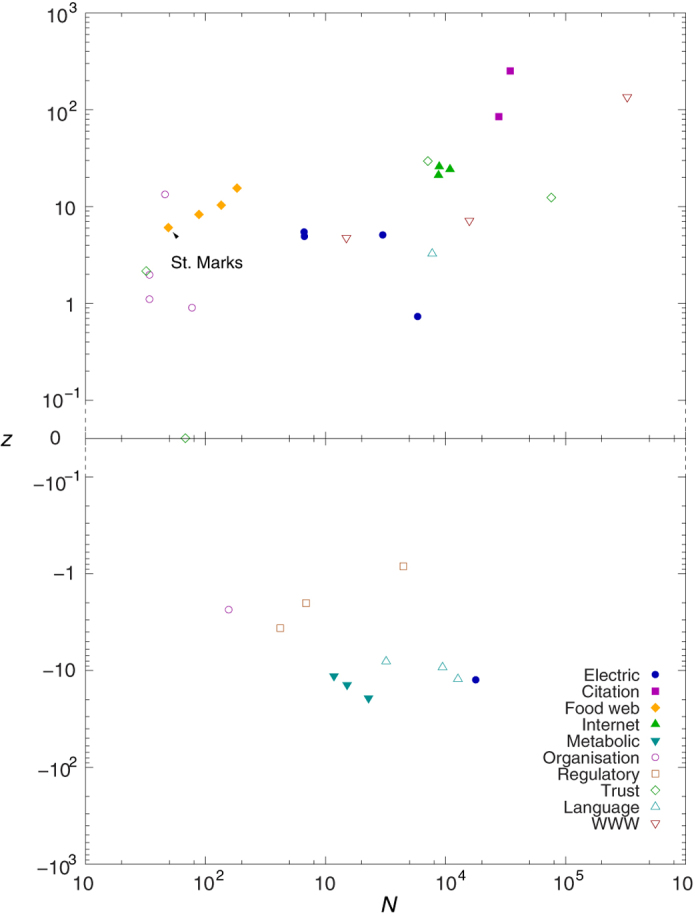
The *z*-score of *H* for the networks shown in Fig. 5. The *z*-score, given by *z* = (*H* − 〈*H*〉)/*σ*(*H*) is plotted as a function of the system size *N*. The random null-model for evaluating 〈*H*〉 and *σ*(*H*) is corresponding to the configuration model.

In Fig. 6 we show the *z*-scores corresponding to the *H* values displayed in Fig. 5 According to the results, the citation networks and the network between the web pages of the nd.edu domain achieve outstandingly high *z*-scores. Furthermore, all of the food webs, the Internet networks and also the rest of the WWW networks obtain considerably large positive *z*-scores. This means that the structure of these networks is far more hierarchical compared to a random network with the same degree distribution. In contrast, all of the regulatory- and metabolic networks have negative *z*-scores, (with rather large absolute values in the latter case). Thus, these networks are less hierarchical compared to what we would expect on a random base. Finally, in case of the electric-, organisational-, and language networks we see a mixed picture, where both positive and negative *z*-scores occur. Most of the organisational networks have positive *z*-scores, reaching to a quite high value in case Consulting network, while in parallel we obtain a negative *z*-score for the Enron network. The word adjacency network for the French, Spanish and Japanese languages have negative *z*-scores, opposed to a clearly positive *z*-score in case of the English language. A more detailed analysis of these results is provided in the Supplementary Information S5. Furthermore, we compare the behaviour of *H* to previously introduced alternative one dimensional hierarchy measures on a part of the studied real systems in Sect.S7.2. in the Supplementary Information. Finally, the robustness of *H* against rewiring of a small number of random links in the networks is examined in Sect.S8. in the Supplementary Information.

## Discussion

Measuring the significance of hierarchical organisation in the structure of a complex network is a non-trivial problem with a number of different options available. Here we have proposed a novel method based on random walks on the network. The basic idea behind our approach is that if nodes were sending instructions or information over the links to their subordinates, then the sources of the information could be traced using random walkers traversing the links backwards. The update rules of the dynamics are chosen in a way to make the density of the walkers on the nodes converge to a stationary distribution exponentially fast with the number of iterations. The position of the nodes in the hierarchy is determined by this distribution, with high random walker densities corresponding to top nodes, and low values of the distribution signalling bottom nodes. The overall measure of the hierarchy is given by the inhomogeneity of the stationary distribution.

The calculation of the hierarchy measure can be carried out based on repeated multiplications of an *N* by *N* transition matrix, making the method computationally very efficient and opens up the possibility for GPU based parallelisation. The other main advantage of our approach is that it can differentiate between directed acyclic graphs of distinct nature, opposed to most other methods treating all directed acyclic networks as already maximally hierarchical. The random walk hierarchy measure provides higher scores for trees showing a multilevel pyramidal structure compared to chains and stars. This is consistent with a general intuitive picture about hierarchies: the multilevel pyramidal structure enables the leaders in the tip of the hierarchy to reach the rest of the system via relatively short paths, and also avoids the ‘overloading’ of any nodes with a too large number of direct subordinates. In contrast, the distance between the top and the bottom becomes very large in a chain, while the number of direct subordinates for the central node is diverging with the system size in case of a star. A further interesting property of our measure is that it is behaving similarly to intensive quantities in physics. I.e., for regular trees with a constant branching number the hierarchy measure is converging to a well defined value in the thermodynamic limit. Thus, above a certain scale it is the structure, (encoded in the branching number), what determines the hierarchy, not the size of the network.

Moreover, our tests on real world networks provided rather encouraging results. On the one hand, the detailed analysis of the St. Marks food web resulted in hierarchy levels that are highly consistent with former results from ecology on the effective trophic levels in the system. On the other hand, the large scale analysis of numerous further real networks revealed that the value of the hierarchy measure on its own does not always provide a fair characterisation of the importance of hierarchy in the structure of the studied system. According to our results, in some cases a relatively low *H* value can be accompanied by an outstandingly high *z*-score, when we compare the actual *H* to the expected value of *H* in a randomly rewired network with the same degree distribution. This leads to the conclusion that the basic network characteristics such as the link density, degree distribution, etc. can inflict some constrains on the possible range of *H* and also on 〈*H*〉 in the corresponding random network ensemble. However, the further analysis of these effects is out of the scope of the present paper and is providing interesting directions for further research.

## Methods

### Hierarchy of chains, regular trees and stars

First we note that *H* = 0 when the network is consisting of only a single directed cycle. Since all the nodes are equivalent in this case, 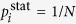 for all *i*, thus, *σ*(**p**^stat^) = 0. Now let us examine how does *H* change if we move from a cycle to a chain by cutting a single link. Since all the nodes have a unit in-degree except the first node, and all the nodes have a unit out-degree except the last node, the transition probability from level *l* to level *l* − 1 is unity. Therefore, the stationary distribution on the last node is zero, 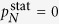, (since the total amount of injected random walkers exit immediately, and there is no inflow of walkers from outside). Based on (8), the stationary distribution of the walkers on the intermediate levels fulfils


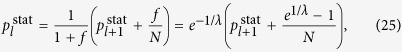


while in case of the first node the injected random walkers cannot exit the node, resulting in


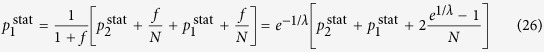


By solving (25–26) we gain


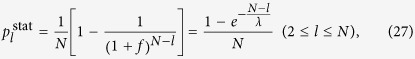



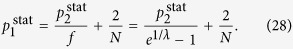


By substituting into (12) the result simplifies to (13).

The random walk hierarchy measure for general trees with varying branching number cannot be given in a general formula, nevertheless it can be calculated exactly for any particular finite tree based on (9) and (12). However, in case of a regular tree with branching number *b*, a simple recursion can be given for the stationary distribution of the random walkers, as the transition probability from any node to its ‘leader’ in the level above is simply 1/*b*. The random walkers cannot exit from the root, which we label as level *l* = 1, and there is an inflow of walkers from the second level, resulting in


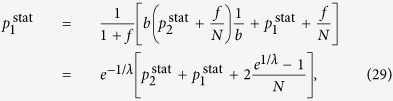


which is exactly the same as in case of the first node in the chain, given in (26). For the intermediate levels, we have an inflow of walkers from the level below, and also a term corresponding to the probability of the walkers staying at the given level instead of moving to the level above, yielding altogether


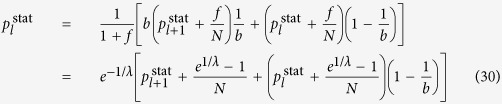


Finally, on the last level *l* = *l*_max_ we have no inflow from other nodes, giving


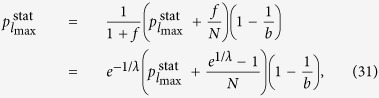


which provides an immediate solution for 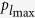 in the form given in (16). Based on (16) we can calculate the stationary distribution on the rest of the levels as well, i.e., by rearranging (29) and (30) we gain (17) and (18).

The hierarchy measure for the star can be evaluated in a similar fashion to that of the chain. In this case *N* − 1 peripheral nodes are connected to a central node, from which the random walkers cannot exit. Thus, the stationary distribution of the random walkers fulfil


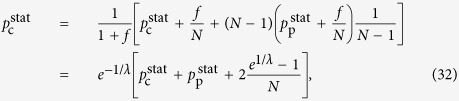



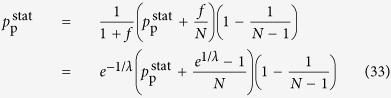


where 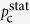 denotes the density on the central node, and 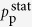 is equal to the density on the peripheral nodes. From (33) we can express 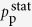 directly as


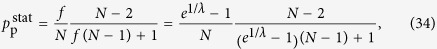


and by substituting (34) into (32) we arrive to


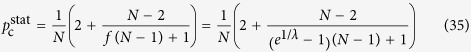


for the central node. According to (12), the random walk hierarchy measure can be given in this case as


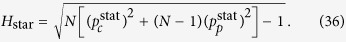


By substituting (34) and (35) into (36), the resulting formula can be simplified to (15).

### Hierarchy in the thermodynamic limit

Taking the *N* → ∞ limit of *H*_chain_ given in (13) and of *H*_star_ written in (15) is trivial, the results are given in (20) and in (22) respectively. In contrast, the evaluating the *N* → ∞ limit of *H*_tree_ given in (14) is more complicated and can be carried out as follows.

First we separate the first term from the rest in the sum over the levels in (14) as


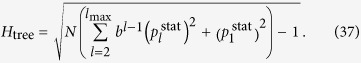


In order to evaluate the remaining sum, we express 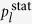 given in (17) as


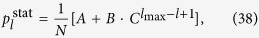


where


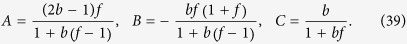


Based on the above,


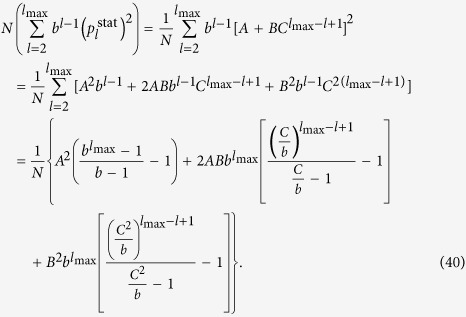


By using that 
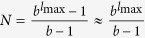
 if *N* ≫ 1, (40) can be also written as


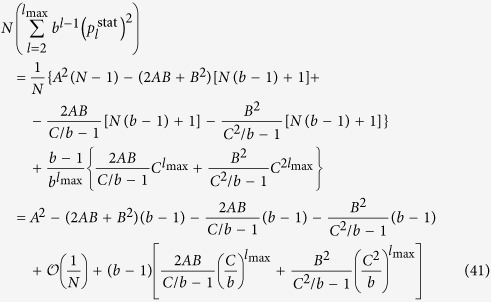


According to (39)


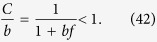


However, the similar inequality of


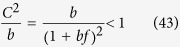


holds if and only


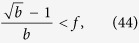


or in terms of *λ* if and and only


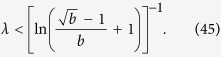


Thus, when (44), or equivalently (45) are fulfilled, the last two terms in (41) vanish if *l*_max_ → ∞. By using (39) and neglecting the 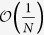 terms we obtain


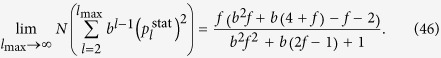


Now let us examine the 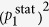 term in (37). According to (29) we can write


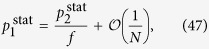


where


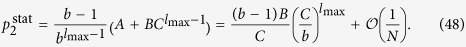


Since 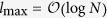 and 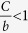,


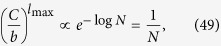


and we obtain that


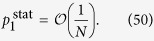


As a consequence, 
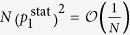
, which is also vanishing when *N* → ∞. Hence, by substituting (46) into (37) and neglecting the 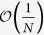 terms we arrive to


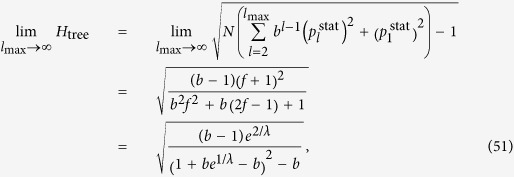


equivalent to the formula given in (21).

### Generating trees with a varying branching number

We used the following algorithm for generating a tree with varying branching number between *N* nodes:

- Initially the nodes are ordered, however, they are also completely isolated from each other.
- We iterate over the nodes according to their order. For current node *i* we draw a number *κ*(*i*) from a Poisson distribution with a fixed parameter *α*, and assign the branching number *b*(*i*) = *κ*(*i*) + 1 to the node. (This way it is guaranteed that the branching number of *i* is *b*(*i*) ≥ 1).
- We scan the nodes coming after *i* and stop at the first node *j* with no incoming link. We attach directed links pointing from *i* to the nodes starting from *j* and ending at *j* + *b*(*i*) − 1.
- We repeat steps (ii)–(iii) until all nodes become connected to the tree.

The advantage of this algorithm is that it enables the study of the swift transition from a chain through a family of trees with increasing average branching number to a star. I.e., if we set the parameter of the Poisson distribution to *α* = 0, we obtain *b*(*i*) = 1 for all nodes, thus, the resulting graph is actually a chain. However, if *α* is large enough compared to *N*, the branching number drawn for the first node is already larger than *N*, thus, we obtain a star. For intermediate parameter values the average branching number of the tree is of course 〈*b*〉 = 1 + *α*. However, the branching numbers of the individual nodes in the tree will deviate from this average in a similar manner to real systems.

## Additional Information

**How to cite this article**: Czégel, D. and Palla, G. Random walk hierarchy measure: What is more hierarchical, a chain, a tree or a star? *Sci. Rep.* **5**, 17994; doi: 10.1038/srep17994 (2015).

## Supplementary Material

Supplementary Information
