# Supplementary material for: Random walk hierarchy measure: What is more hierarchical, a chain, a tree or a star?
Source: Sci Rep. 2015 Dec 10;5:17994. doi: 10.1038/srep17994 (PMC4675073; doi:10.1038/srep17994)
Supplement: Supplementary Information [file srep17994-s1.pdf]

Random walk hierarchy measure: What is more hierarchical, a chain, a tree or a star? Supplementary Information

Dániel Czégel[1] and Gergely Palla[2,3*]

[1]Dept. of Biological Physics, Eötvös University, H-1117 Budapest, Hungary

[2]MTA-ELTE Statistical and Biological Physics Research Group, Hungarian Academy of Sciences, H-1117 Budapest, Hungary,

[3]Regional Knowledge Centre, Eötvös University, H-8000 Székesfehérvár, Hungary,

October 1, 2015

S1 Measuring the level of hierarchical organisation in a network

Characterising the importance of hierarchy in a given network structure is a non-trivial problem with a large number of alternative approaches. Without loss of generality we can formulate a few intuitive requirements a hierarchy measure should meet: First, we assume no a priori ordering between the nodes, the measure is evaluated purely based on the topology of the network. Moreover, the hierarchy measure should should not be too sensitive to the local structure of the network, i.e., the degree sequence alone should not provide enough information for complete evaluation of H. In other words, we should be able to modify H when rewiring the links in a network without changing the degree sequence. E.g., let us suppose that the network is corresponding to a single directed cycle of N nodes, (a giant directed "ring"). Since all the nodes are equivalent, the network lacks any hierarchy what so ever, thus, H should be equal to 0. However, if we take just a single link away, (which is only a minor change if N is large), the network becomes a directed chain, which is indeed hierarchical, thus, a significant jump should be observed in H.

*Corresponding author, e-mail: pallag@hal.elte.hu

One of the first hierarchy measure was proposed by D. Krackhardt, motivated by organisational hierarchy [1]. The main assumption here is that in a hierarchical company we can reach the lower levels of the hierarchy from the levels above via chains of commands, and in contrast, we cannot reach the higher levels in a similar fashion from the levels below. Thus, on the one hand, we count the total number of ordered pairs (i, j) in the network for which there is directed path either from i to j or from j to i, but not both. On the other hand, we also evaluate the total number of connected pairs, for which at least one directed path exists between the two nodes, (but we also allow paths in both directions). By denoting the number of ordered pairs by A, and the number of connected pairs by C, Krackhardt's hierarchy measure [1] is simply given by

$$H_{\rm K} = \frac{A}{C}. \tag{S1}$$

Based on the definition above, $H_K = 1$ for all acyclic networks, where all the node pairs are ordered. Nevertheless, H_K is a fine example for a hierarchy measure depending mostly on the global structure of the network: For a directed cycle of N nodes $H_K = 0$, (since we can reach any node from any other node, and hence, every pair is unordered). By deleting a single link we turn the network into a directed chain, (where all the pairs are ordered), and suddenly the hierarchy measure jumps to $H_K = 1$.

An approach motivated by similar intuitions compared to Krackhard's hierarchy was introduced by E. Mones et al., assuming that reaching the rest of the network should be relatively easy for the nodes high in the hierarchy, and more difficult for the nodes at the bottom of the hierarchy [2]. Here the position of the node i in the hierarchy is determined by its reaching centrality, $C_{\rm R}(i)$, corresponding to the fraction of nodes that can be reached from i, (following directed paths). Based on the $C_{\rm R}$ associated to the individual nodes, the Global Reaching Centrality of the whole network is defined as [2]

$$H_{\rm GRC} = \frac{\sum_{i=1}^{N}\left[C_{\rm R}^{\rm max} - C_{\rm R}(i)\right]}{N-1}, \tag{S2}$$

where the summation is running over the nodes, the size of the network is given by N and the maximal reaching centrality is denoted by $C_{\rm R}^{\rm max}$.

The maximal possible value of this hierarchy measure is $H_{\rm GRC} = 1$, which is obtained when the network is corresponding to a star with $N-1$ arms, with only the central node having a non zero reaching centrality. Interestingly, for a chain of N nodes, we obtain $H_{\rm GRC} = 1/2 \cdot N/(N-1)$,

which is still larger than 1/2. Similarly to the previous measure, $H_{\rm GRC} = 0$ for a directed cycle.

Another way for quantifying the possible asymmetry between nodes is to assume some sort of flow on the links, and examine whether the global map of flows in the system is revealing a kind of overall directionality or not. Probably the simplest approach in this framework is to define the fraction of links not participating in any cycle as the measure of the hierarchy. I.e., the link flow hierarchy proposed by J. Luo and C. L. Magee can be formulated as [3]

$$H_{\rm LF} = \frac{M_{\rm ac}}{M}, \tag{S3}$$

where $M_{\rm ac}$ denotes the number of acyclic links, not part of any directed cycle, and M is corresponding to the total number of links in the network.

Similarly to Krackhardt's hierarchy, the link flow hierarchy is $H_{\rm LF} = 1$ for all acyclic networks. Furthermore, when the network is corresponding to a single directed cycle, $H_{\rm LF} = 0$. Thus, when deleting a link from this cycle, we observe a jump from zero hierarchy to maximal hierarchy.

A more elaborate quantification of hierarchy was proposed by B. Corominas-Murta et al. [4] with the introduction of treeness, feedforwardness and orderability, projecting the studied network onto a point in a 3 dimensional space, where each dimension is aimed to capture a different aspect of hierarchy. Treeness, T, is measuring how ambiguous are the chain of commands in the network. I.e., in a regular tree where links are pointing from higher levers to lower levels we obtain $T = 1$, whereas if revert the link directions, the obtained structure is considered anti-hierarchical, with $T = -1$. The calculation of T is based on comparing forward and backward entropies [4].

In the mean time Feedforwardness, F is related to the size and position of the strongly connected components in the network. Since we can reach any node from any other node in a strongly connected component, we cannot define an ordering amongst these nodes. Furthermore, if the strongly connected component is found near the top of the overall hierarchy, its effect is more severe compared to a situation where it is occurring only at the deeper levels.

Finally, the orderability, O is simply the fraction of nodes not taking part in any directed cycles,

$$O = N_{\rm ac}/N, \tag{S4}$$

where $N_{\rm ac}$ is the number of nodes that are not members in any directed cycles, and N is the total number of nodes. Thus, orderability is analogous

to the link-flow hierarchy H_{LF} given in Eq.(S3), the only difference is that here we measure the weight of the cycles in the network by the number of contained nodes instead of the number of contained links. Similarly to H_{LF}, the oredrability is $O = 1$ for all acyclic networks, while $O = 0$ when the network is corresponding to a single directed cycle.

S2 Alternative inhomogeneity measure

An alternative possibility for measuring the inhomogeneity of the stationary distribution of the random walkers is to average the difference between the maximum of the distribution and p_i^{stat}. This idea is used for example in the definition of the Global Reaching Centrality given in Eq.(S2), where H_{GRC} is obtained from averaging the difference between the reach of the individual nodes and the maximal reach in the system. In our case, the hierarchy measure would read

$$\widehat{H} = \frac{\sum_{i=1}^{N} \left[\max(p^{\mathrm{stat}}) - p_i^{\mathrm{stat}}\right]}{N-1} \tag{S5}$$

in this approach, where $\max(p^{\mathrm{stat}})$ denotes the largest random walker density observed in the network.

One of the drawbacks of Eq.(S5) is that a single outlier in the distribution can induce a large change in the hierarchy value. The other problem with $\widehat{H}$ is that it is converging to zero for large regular trees, (in contrast to H defined in the main paper, providing a finite non-zero hierarchy value even for infinitely large regular trees). This is demonstrated in Fig.S1., where we plotted $\widehat{H}$ as a function of the system size for chains, regular trees and stars, in a similar fashion to Fig.1. in the main paper, (showing the behaviour of the original random walk hierarchy measure H). According to Fig.S1., the $\widehat{H}$ values obtained for a chain and a star are almost indistinguishable, showing a power-law like decay as a function of N. Although the $\widehat{H}$ calculated for regular trees is clearly higher, it is also decaying as a power-law, only the magnitude of the decay exponent is smaller.

S3 Intensiveness

We have seen on Fig.1. in the main paper that H of finite regular trees is converging to the limit value for infinitely large trees already around $N = 1000$ when $\lambda = 2$, while the convergence is somewhat slower when $\lambda = 4$. Nevertheless, above a certain N it is the structure of the tree,

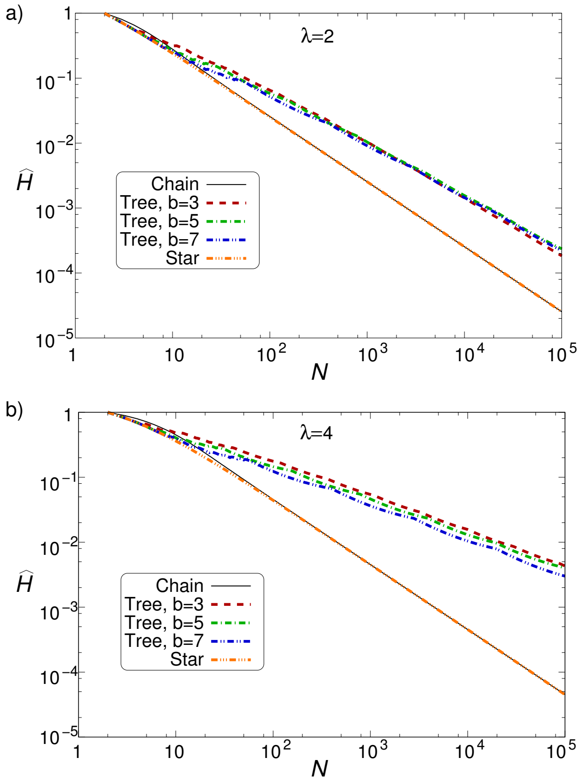

Figure S1: The behaviour of $\widehat{H}$, defined in Eq.(S5), for chains, trees and stars.

(encoded in the branching number), what determines the hierarchy measure, not the size of the tree. This indicates that H is behaving similarly to intensive quantities in physics in some aspects.

To investigate this property further, let us examine what happens to H if we take a pair of disjoint graphs $\mathcal{G}_1$ and $\mathcal{G}_2$, and unite them into a single graph $\mathcal{G}_1 \cup \mathcal{G}_2$ with two isolated components. According to Eq.(10) in the main paper, the hierarchy measure of the graphs when considered separately can be written as

$$H_{\mathcal{G}_1} = \sqrt{N_1 \sum_{i=1}^{N_1} (p_i^{\rm stat})^2 - 1}, \tag{S6}$$

$$H_{\mathcal{G}_2} = \sqrt{N_2 \sum_{j=1}^{N_2} (p_j^{\rm stat})^2 - 1}, \tag{S7}$$

where $p_i^{\rm stat}$ and $p_j^{\rm stat}$ correspond to the stationary distribution of the random walkers on nodes $i \in \mathcal{G}_1$ and $j \in \mathcal{G}_2$, while N_1 and N_2 denote the sizes of $\mathcal{G}_1$ and $\mathcal{G}_2$ respectively. When considering the union of the two graphs, the stationary distribution of the random walkers has to be normalised over the whole range of $N_1 + N_2$ nodes. Thus, the stationary distribution on node i, originally in $\mathcal{G}_1$, now part of $\mathcal{G}_1 \cup \mathcal{G}_2$, can be given as $\tilde{p}_i^{\rm stat} = p_i^{\rm stat} N_1/(N_1 + N_2)$. Likewise, the stationary distribution on node j, originally in $\mathcal{G}_2$, now part of $\mathcal{G}_1 \cup \mathcal{G}_2$, can be expressed as $\tilde{p}_j^{\rm stat} = p_j^{\rm stat} N_2/(N_1 + N_2)$. Therefore, the hierarchy measure of the union of the two graphs reads

$$\begin{aligned} H_{\mathcal{G}_1 \cup \mathcal{G}_2} &= \sqrt{(N_1+N_2)\left(\sum_{i=1}^{N_1}(\tilde{p}_i^{\rm stat})^2 + \sum_{j=1}^{N_2}(\tilde{p}_j^{\rm stat})^2\right) - 1} = \\ & \sqrt{\frac{N_1^2 \sum_{i=1}^{N_1}(p_i^{\rm stat})^2 + N_2^2 \sum_{j=1}^{N_2}(p_j^{\rm stat})^2}{N_1+N_2} - 1}. \end{aligned} \tag{S8}$$

By rearranging Eqs.(S6-S7) we gain

$$\sum_{i=1}^{N_1}(p_i^{\rm stat})^2 = \frac{[H_{\mathcal{G}_1}]^2+1}{N_1}, \tag{S9}$$

$$\sum_{i=1}^{N_1}(p_i^{\rm stat})^2 = \frac{[H_{\mathcal{G}_1}]^2+1}{N_2}, \tag{S10}$$

and by substituting Eqs.(S9-S10) into Eq.(S8) we obtain

$$H_{\mathcal{G}_1 \cup \mathcal{G}_2} = \sqrt{\frac{N_1\,[H_{\mathcal{G}_1}]^2 + N_2\,[H_{\mathcal{G}_1}]^2}{N_1+N_2}}. \tag{S11}$$

Thus, the hierarchy measure of the union of two isolated networks is simply the weighted quadratic mean of H obtained for the individual graphs. A noteworthy consequence is that if $H_{\mathcal{G}_1} = H_{\mathcal{G}_2}$, then this implies also that the hierarchy measure of the union is also the same, $H_{\mathcal{G}_1 \cup \mathcal{G}_2} = H_{\mathcal{G}_1} = H_{\mathcal{G}_2}$. Furthermore, according to Eq.(S11) in general, if we take the union of $\mathcal{G}_1$ and $\mathcal{G}_2$ which are isolated from each other and $H_{\mathcal{G}_1} \leq H_{\mathcal{G}_2}$, then the hierarchy measure of the resulting network will be between the hierarchies measured for the two graphs separately,

$$H_{\mathcal{G}_1} \leq H_{\mathcal{G}_1 \cup \mathcal{G}_2} \leq H_{\mathcal{G}_2}. \tag{S12}$$

Therefore, the rule for calculating H for a system composed of isolated smaller parts based on H obtained for these sub-systems is showing again a great deal of similarity to the behaviour of intensive physical quantities.

S4 The trees of maximal hierarchy

According to our results in the main paper, when considering regular trees of infinitely large size, the hierarchy measure may either diverge or remain finite, depending on both the branching number b and the value of the λ parameter. At a fixed b, the critical $\lambda_c(b)$ separating the two regimes is given by Eq.(23) in the main paper. Here, in Fig.S2. we show λ_c as a function of b on a semi-logarithmic plot, where the regime of diverging H values is coloured grey.

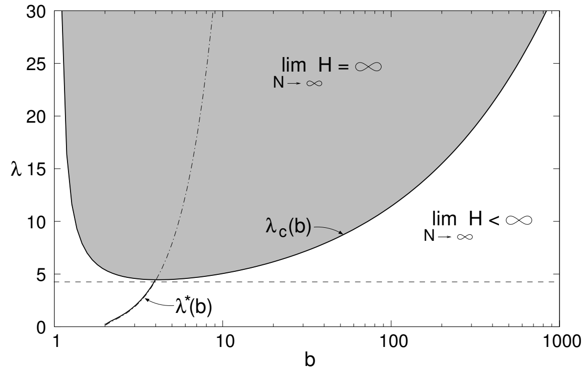

Figure S2: The "phase diagram" for H in case of regular trees in the thermodynamic limit. If λ and b are falling into the region shown in grey, (meaning that λ is larger than $\lambda_c(b)$ calculated from Eq.(23) in the main paper), then H is diverging. In contrast, for parameter settings falling in the white region, H remains finite in the thermodynamic limit. The maximal H in this region is obtained at a $\lambda^*(b)$, given in Eq.(S14). The dashed horizontal line is showing the minimum of $\lambda_c(b)$.

In the regime of finite H, the limit value for the hierarchy measure is given by Eq.(21) in the main paper. The maximum of this function can be located by solving

$$b^2(e^{1/\lambda}-1)-2b(e^{1/\lambda}-1)-2=0. \tag{S13}$$

Based on that, at a fixed branching number b, the λ parameter providing

the maximal H can be written as

$$\lambda^* = \left[\ln\left(\frac{2}{b^2-2b}+1\right)\right]^{-1}, \tag{S14}$$

while at a fixed λ parameter the tree with maximal hierarchy has a branching number of

$$b^* = 1 + \frac{\sqrt{e^{2/\lambda}-1}}{e^{1/\lambda}-1}. \tag{S15}$$

In Fig.S2. we also show λ^* given in Eq.(S14) as a function of b. In parallel, the b^* expressed in Eq.(S15) is plotted as a function of λ in Fig.S3. According

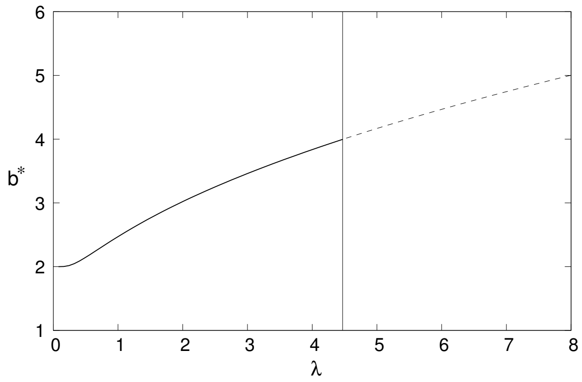

Figure S3: The branching number b^* for which H is maximal, as a function of the parameter λ in case of infinitely large regular trees. The vertical line is corresponding to the minimum of $\lambda_c(b)$ given in Eq.(23) in the main paper, thus, for λ values above that H can become divergent in the thermodynamic limit.

to picture, the maximum of H is at $b = 2$ when λ is low. However, b^* is steadily increasing as a function of λ, reaching to $b^* \approx 3.84$ at $\lambda = 4$, chosen to be the optimal λ value based on arguments given in the main paper.

S5 Hierarchy of real networks

We have analysed the hierarchy in a large number of networks of different types and varying sizes, the scatter plot of the obtained H values and the z-score of H are given in Figs.5-6. in the main paper. In Tables S1-S2. we provide the results in more details by listing also the size and the average degree beside the H values and z-scores of the examined networks.

Type	Meaning of $A \to B$	Network	N	$\langle k \rangle = \frac{M}{N}$	H
Electric	B depends on	s1488 [5]	667	2.085	0.893
	the value at A	s1494 [5]	661	2.116	0.880
		s5378 [5]	2993	1.467	0.887
		s9234 [5]	5844	1.400	0.870
		s35932 [5]	17828	1.683	0.719
Citation	A is cited by B	ArXiv-HepPh [6]	34546	12.203	0.352
		ArXiv-HepTh [6]	27770	12.705	0.430
Food web	A eats B	GrassLand [7]	88	1.557	1.551
		LittleRock [8]	183	13.628	0.482
		St. Marks [9]	49	4.612	0.634
		Ythan [7]	135	4.452	0.677
Internet	A sent messages	p2p-1 [10]	10876	3.677	0.524
	to B	p2p-2 [10]	8846	3.599	0.541
		p2p-3 [10]	8717	3.616	0.529
Metabolic	B is an end	C. elegans [11]	1173	2.442	0.467
	product of A	E. coli [11]	2275	2.533	0.447
		S. cerevisiae [11]	1511	2.537	0.440
Organization	B trusts in A	Consulting [12]	46	19.109	0.120
		Enron [13]	156	10.699	0.126
		Manufacturing [12]	34	18.935	2.18×10^{-2}
	B knows A	Freemans-1 [14]	34	18.971	6.35×10^{-2}
		Freemans-2 [14]	77	24.412	1.06×10^{-2}
Regulatory	A regulates B	TRN-Yeast-1 [15]	4441	2.899	0.262
		TRN-Yeast-2 [16]	688	1.568	0.720
		TRN-EC [16]	419	1.239	0.823
Trust	B trusts in A	College [17]	32	3.000	0.464
		Epinions [18]	75888	6.705	0.437
		Prison [19]	67	2.716	0.565
		WikiVote [20]	7115	14.573	0.201
Language	word B	English [21]	7724	5.992	0.404
	follows word A	French [21]	9424	2.578	0.478
		Spanish [21]	12642	3.570	0.194
		Japanese [21]	3177	2.613	0.497
World Wide	B has a link to A	Google web [22]	15763	10.861	0.258
Web		nd.edu [23]	325729	4.596	0.557
		Polblogs [24]	1490	12.812	0.223

Table S1: Random walk hierarchy of real networks shown in Fig.5. in the main paper. The network type is given in the 1st column, the meaning of the links in the 2nd column, the references to the data sources are listed in the 3rd column. The network size is given in the 4th column, followed by the average degree in the 5th column. The hierarchy measure H (calculated at the optimal $\lambda = 4$ parameter value) is provided in the 6th column.

The electric networks, where the target of a directed link is depending on

the value of the source node, turned out to be rather hierarchical according to our measure. Although their sizes is varying between $N = \mathcal{O}(10^2)$ and $N = \mathcal{O}(10^4)$, the hierarchy values are quite close to each other, forming an elongated cluster in Fig.5. in the main paper. The analysed two citation networks, where the links are pointing from the cited paper to the citing article, showed only a moderate amount of hierarchy. In contrast, a part of the food webs were amongst the most hierarchical networks in the studied examples. However, the H values showed a relative large variance for this network type, which can be due to the different habitat of the species involved in the listed food webs. Furthermore, in case of the St. Marks food web the links were weighted, with the weights corresponding to the fractions of the consumers diet, whereas in the other cases the links were un-weighted.

The peer to peer message networks over the Internet and the metabolic networks, (where the target of a link was corresponding to a product of the source) showed moderate levels of hierarchy. For both types, the data points in Fig.5. in the main paper formed tight clusters. The intra organisational networks were the least hierarchical according to our measure, with the data points forming a cluster close to the origin. Similarly to the food webs, the variance of the H values obtained for the regulatory networks was relatively high. I.e., the regulatory networks TRN-Yeast-2 and TRN-Yeast-1 are amongst the most hierarchical studied systems, whereas the TRN-EC network shows only a modest hierarchy value.

The trust networks obtained moderate hierarchy values, and the variance of their H values is far smaller compared to the very large variance in their sizes. The language networks turned out to be moderately hierarchical as well. Here the networks were originating from large text corpora, and a directed link from word A to B is signaled that B was following A in the text. According to the results, the English- French- and Japanese language networks obtained H values quite close to each other, while the hierarchy of the Spanish language seemed considerably lower. Finally, the networks between web pages showed again a moderate hierarchy, where H for the Google web and Polblogs were quite close to each other, while network of nd.edu received a significantly higher hierarchy value.

It is very interesting to see how the picture is changing when we switch from H to the z-score shown in Fig.6 in the main paper, corresponding to the difference between H and $\langle H \rangle$ in the configuration model, scaled by standard deviation of H in the configuration model. The citation networks, the Internet networks and the food webs are forming three rather tight clusters in Fig.6. in the main paper with significant positive z-score, (which is outstandingly high in case of the citation networks). In contrast, the metabolic-

Type	Network	H	$\langle H_{\mathrm{rand}} \rangle \pm \sigma(H_{\mathrm{rand}})$	z
Electric	s1488 [5]	0.893	$0.811 \pm 1.66 \cdot 10^{-2}$	4.94
	s1494 [5]	0.880	$0.780 \pm 1.83 \cdot 10^{-2}$	5.46
	s5378 [5]	0.887	$0.803 \pm 1.65 \cdot 10^{-2}$	5.09
	s9234 [5]	0.870	$0.861 \pm 1.23 \cdot 10^{-2}$	0.73
	s35932 [5]	0.719	$0.787 \pm 5.44 \cdot 10^{-3}$	-12.5
Citation	ArXiv-HepPh [6]	0.352	$0.222 \pm 5.16 \cdot 10^{-4}$	251.94
	ArXiv-HepTh [6]	0.430	$0.221 \pm 2.46 \cdot 10^{-3}$	84.96
Food web	GrassLand [7]	1.551	$1.036 \pm 6.20 \cdot 10^{-2}$	8.31
	LittleRock [8]	0.482	$0.221 \pm 1.68 \cdot 10^{-2}$	15.54
	St. Marks [9]	0.634	$0.425 \pm 3.44 \cdot 10^{-2}$	6.08
	Ythan [7]	0.677	$0.450 \pm 2.19 \cdot 10^{-2}$	10.37
Internet	p2p-1 [10]	0.524	$0.488 \pm 1.48 \cdot 10^{-3}$	24.32
	p2p-2 [10]	0.541	$0.499 \pm 1.62 \cdot 10^{-3}$	25.92
	p2p-3 [10]	0.529	$0.495 \pm 1.61 \cdot 10^{-3}$	21.12
Metabolic	C. elegans [11]	0.467	$0.611 \pm 1.20 \cdot 10^{-2}$	-12.00
	E. coli [11]	0.447	$0.630 \pm 8.99 \cdot 10^{-3}$	-20.36
	S. cerevisiae [11]	0.440	$0.609 \pm 1.14 \cdot 10^{-2}$	-14.82
Organization	Consulting [12]	0.120	$4.99 \cdot 10^{-2} \pm 5.24 \cdot 10^{-3}$	13.38
	Enron [13]	0.126	$0.160 \pm 1.44 \cdot 10^{-2}$	-2.36
	Manufacturing [12]	2.18×10^{-2}	$2.04 \cdot 10^{-2} \pm 1.27 \cdot 10^{-3}$	1.10
	Freemans-1 [14]	6.35×10^{-2}	$6.01 \cdot 10^{-2} \pm 1.72 \cdot 10^{-3}$	1.98
	Freemans-2 [14]	1.06×10^{-2}	$9.42 \cdot 10^{-3} \pm 1.20 \cdot 10^{-3}$	0.98
Regulatory	TRN-Yeast-1 [15]	0.262	$0.264 \pm 2.39 \cdot 10^{-3}$	-0.84
	TRN-Yeast-2 [16]	0.720	$0.733 \pm 6.45 \cdot 10^{-3}$	-2.02
	TRN-EC [16]	0.823	$0.853 \pm 8.22 \cdot 10^{-3}$	-3.65
Trust	College [17]	0.464	$0.403 \pm 2.82 \cdot 10^{-2}$	2.16
	Epinions [18]	0.437	$0.373 \pm 5.15 \cdot 10^{-3}$	12.43
	Prison [19]	0.565	$0.565 \pm 4.94 \cdot 10^{-2}$	0
	WikiVote [20]	0.201	$0.157 \pm 1.49 \cdot 10^{-3}$	29.53
Language	English [21]	0.404	$0.388 \pm 4.89 \cdot 10^{-3}$	3.27
	French [21]	0.478	$0.519 \pm 4.64 \cdot 10^{-3}$	-8.83
	Spanish [21]	0.194	$0.241 \pm 4.03 \cdot 10^{-3}$	-11.66
	Japanese [21]	0.497	$0.560 \pm 8.24 \cdot 10^{-3}$	-7.65
World Wide	Google web [22]	0.258	$0.246 \pm 1.68 \cdot 10^{-3}$	7.14
	nd.edu [23]	0.557	$0.451 \pm 7.84 \cdot 10^{-4}$	135.20
	Polblogs [24]	0.223	$0.184 \pm 8.21 \cdot 10^{-3}$	4.75

Table S2: Comparing H in real networks to that of their link randomised counterparts. The network type is given in the 1st column, the references to the data sources are listed in the 2nd column, and the H measured in the original networks is given in the 3rd column. The average value of H in the configuration model, (calculated via link randomisation) is presented in the 4th column, accompanied by the standard deviation. Finally, the corresponding z-scores are listed in the 5th column.

and regulatory networks are forming clusters of significant negative z-score, indicating that their structure is far less hierarchical compared to what we would expect by assuming random connections between the nodes, (at the same degree distribution as in the original network). The z-scores of WWW networks were all positive, (where the nd.edu network showed an outstandingly high value). In case of the trust networks the z-scores are also almost always positive, with the exception of the Prison network, where $z = 0$. The organisational-, electric-, and language networks showed a mixed picture, including both positive and negative z-scores.

S6 Correlations with PageRank and reaching centrality

Since our hierarchy measure is based on random walks on the network structure, it is quite natural to ask what is the relation between PageRank [25] and the stationary distribution of random walkers in our model? It is well known that PageRank is also very closely related to random walks on the network, thus, we expect a strong correlation between the two quantities. However, a very important feature of our model is that the transition probability between a pair of connected nodes depends also on the degree of the endpoint, not solely on the degree of the starting point. Based on that, some differences are also expected between the ranking of the nodes according to the PageRank, and the ranking of the nodes according to p_i^{stat}. Another important difference between our model and conventional random walk models on networks is that the random walkers are traversing the links backwards, seeking the source of information. Thus, the direction of the links has to be inverted before the calculation of the PageRank in order to make a fair comparison with p_i^{stat}.

In Table.S3. we show the correlation between p_i^{stat}, (corresponding to the stationary distribution of random walkers in our model), and the PageRank of the nodes, (obtained with inverted link directions), for one system from each main network type studied in the section Results on real networks in the main paper, and also in Sect.S5. According to the results, the compared quantities are positively correlated. However, in the mean time the magnitude of the correlation coefficients are varying to a large extent. For example, in case of the organisational network of Enron both the Pearson's correlation coefficient and the Spearman's rank correlation coefficients are very close to zero. In contrast, for the GrassLand food web we can observe a very strong correlation between p_i^{stat} and the PageRank.

Type	Network	C_{Pearson}	C_{Spearman}
Electric	s5378 [5]	0.547	0.704
Citation	ArXiv-HepTh [6]	0.155	0.681
Food web	GrassLand [7]	0.939	0.745
Internet	p2p-1 [10]	0.704	0.872
Metabolic	E. coli [11]	0.050	0.299
Organization	Enron [13]	0.025	0.028
Regulatory	TRN-Yeast-1 [15]	0.538	0.551
Trust	WikiVote [20]	0.458	0.787
Language	English [21]	0.035	0.149
WWW	Google web [22]	0.368	0.424

Table S3: Correlation between the PageRank and the stationary distribution of random walkers p_i^{stat} in our model. The direction of the links were inverted when calculating the PageRank, and the damping factor was set to the widely used $\alpha = 0.85$ value. The network type is given in the 1st column, the references to the data sources are listed in the 2nd column. The Pearson's correlation coefficient is given in the 3rd column, and the Spearman's rank correlation coefficient is displayed in the 4th column.

In order to give more insight into the relation between the PageRank and the stationary distribution of the random walkers in our model, in Figs.S4-S5. we show the scatter plot of the PageRank as a function of p_i^{stat}. In case of the E. coli network [11] displayed in Fig.S4. the two quantities show very little dependency on each other, and accordingly, the the corresponding correlation values given in Table.S3. are low. In contrast, the points in Fig.S5. corresponding to the Google web [22] show a roughly increasing tendency, accompanied by moderate correlation values in Table.S3.

Based on the above, our conclusion is that the relation between the PageRank and p_i^{stat} shows large variations over the investigated systems. In some examples the two quantities can be quite strongly correlated, while we have also seen networks where they show very small inter dependency. According to that, with the introduction of our random walk based model we do not 'reinvent' PageRank, instead we measure quantities that can behave quite distinctly compared to PageRank.

Finally, we note that a simple hierarchy measure can also be defined based on PageRank in a very similar approach to what we have presented in the section Random walk hierarchy measure in the main paper for our hierarchy measure. The basic idea is that the PageRank of the nodes in a network can be interpreted as a sort of importance or centrality, and the higher PageRank values correspond to higher positions in the hierarchy. The overall hierarchy of a network can be judged based on the inhomogeneity

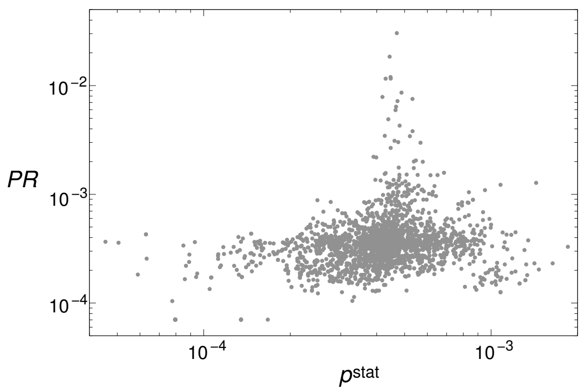

Figure S4: Scatter plot of the PageRank, PR, as a function of the stationary distribution of the random walkers, $p_i^{\rm stat}$, in our model for the E. coli network [11]. The PageRank was calculated for inverted link directions at a damping factor of $\alpha = 0.85$.

of the PageRank distribution, where homogeneous PageRank distributions correspond to non-hierarchical networks, and very inhomogeneous PageRank distributions signal strongly hierarchical systems. Therefore a plausible definition of a hierarchy measure based on the PageRank PR can be given as

$$H_{\rm PR} = \frac{\sigma(PR)}{\mu(PR)}, \tag{S16}$$

where $\mu(PR)$ and $\sigma(PR)$ denote mean and the standard deviation of the PageRank in the network respectively. However, we again draw the attention to the careful treatment of the link directions when using the above formula. In the traditional depicting of hierarchies the links are pointing from nodes higher in the hierarchy towards nodes lower in the hierarchy, and probably the purest example of a hierarchy is given by a regular out-tree with a constant branching number. In order to obtain a higher PR values at the top levels compared to the bottom levels in such systems, the link directions have to be reversed before the evaluation of the PageRank.

S7 Comparison between the hierarchy measures

In this section we compare the random walk hierarchy measure defined in the main paper with previously introduced one dimensional hierarchy measures from the literature. Since the main goal of our work is to develop a simple hierarchy measure preferring trees over chains and stars, our main focus is

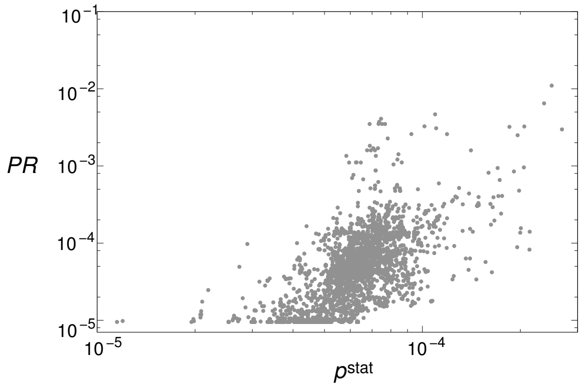

Figure S5: The same plot as in Fig.S4. Google web network [22], showing the PageRank PR as a function of p_i^{stat}. The PageRank was calculated for inverted link directions at a damping factor of $\alpha = 0.85$.

on the examination of the behaviour of the involved measures on acyclic graphs. Therefore, in Sect.S7.1. we reproduce the analysis carried out in the Hierarchy of acyclic networks section in the main paper for Krackhard's hierarchy measure, the Global Reaching Centrality, the link flow hierarchy and also for the hierarchy measure obtained from the inhomogeneity of the PageRank distribution given in Sect.S6. This is followed by the comparison of the hierarchy measures on a couple of real networks in Sect.S7.2.

S7.1 Comparing the hierarchy of acyclic graphs

According to Fig.1. in the main paper, the random walk hierarchy measure does indeed assign higher hierarchy values to trees compared to chains or stars. For comparison, here we examine the behaviour of the alternative one dimensional hierarchy measures from this point of view.

Krackhard's hierarchy [1], given in Eq.(S1), treats all acyclic graphs as already maximally hierarchical, thus, $H_{\rm K} = 1$ for trees and also for both chains and stars, independent of the system size. Therefore, a figure equivalent to Fig.1. in the main paper for Krackhard's hierarchy would be trivial, with constant lines at $H_{\rm K} = 1$ for chains, trees and also stars. The same applies for the link flow hierarchy [3] given in Eq.(S3): since none of the links is taking part in any cycles in a tree, a star, or a chain, the value of the hierarchy measure is $H_{\rm LF} = 1$ for all of these graphs, independent of the system size. Therefore, the figure analogous to Fig.1. is trivial also for $H_{\rm LF} = 1$, consisting of constant lines at $H_{\rm LF} = 1$ for all acyclic graphs.

The Global Reaching Centrality [2], given in Eq.(S2) shows a more complicated behaviour compared to the previous examples. In Fig.S6. we display H_{GRC} as a function of the network size for chains, trees and stars, (in complete analogy with Fig.1. in the main paper for the random walk hierarchy). According to the results, H_{GRC} for chains is showing a decaying tendency, starting at $H_{\mathrm{GRC}} = 1$ for $N = 2$ and converging to $H_{\mathrm{GRC}} = 0.5$ when $N \to \infty$. In contrast, $H_{\mathrm{GRC}} = 1$ for a star independent of N, as pointed out already in Ref.[2]. For regular trees, $H_{\mathrm{GRC}} = 1$ as long as $N \leq b$, where b denotes the branching number. In the $N > b$ regime we can observe first a decay in H_{GRC}, reaching a minimum quite rapidly, and switching to an increasing tendency for large N, converging to $H_{\mathrm{GRC}} = 1$ when $N \to \infty$. Thus, H_{GRC} is maximal for a star at any system size.

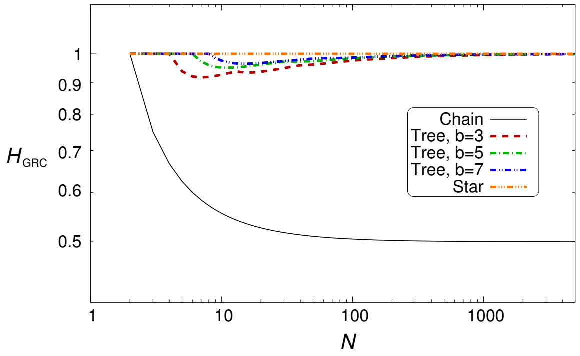

Figure S6: Comparing H_{GRC} for chains, regular trees and stars. The H_{GRC} calculated for chains (black line), regular trees with a branching number $b = 3$ (red line), regular trees with $b = 5$ (green line), regular trees with $b = 7$ (blue line) and stars (orange line) is plotted as functions of the system size N.

Finally, in Fig.S7. we also show the behaviour of H_{PR}, given in Eq.(S16) for acyclic graphs in the same fashion as in Fig.1. in the main paper for the random walk hierarchy. According to the results, H_{PR} is maximal for the star configuration in the entire range of N, showing a power-law-like increasing tendency in the large N regime. In contrast, we can observe a power-law-like decreasing tendency in case of chains. The H_{PR} for regular trees is in-between these two extremes, with a power-law-like increasing tendency, but with a smaller exponent compared to the star configuration.

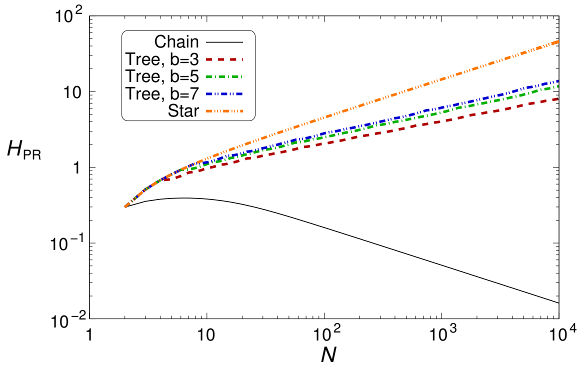

Figure S7: Comparing H_{PR}, (based on the inhomogeneity of the PageRank), for chains, regular trees and stars. The H_{PR} calculated for chains (black line), regular trees with a branching number $b = 3$ (red line), regular trees with $b = 5$ (green line), regular trees with $b = 7$ (blue line) and stars (orange line) is plotted as function of the system size N.

S7.2 Comparing the hierarchy of real networks

In this section we compare the one dimensional hierarchy measures on real networks. We have evaluated Krakhardt's hierarchy, H_{K}, the Global Reaching Centrality, H_{GRC}, the link flow hierarchy, H_{LF}, and the hierarchy measure based on PageRank, H_{PR}, for the same subset of real networks used in Sect.S6. The obtained hierarchy values, together with the random walk hierarchy measure, H, are listed in Table.S4. The results show reasonable similarities between the compared hierarchy measures with interesting differences. For example, all approaches agree on that the Enron network can be considered the least hierarchical among the investigated systems. In contrast, there is a slight disagreement in choosing the most hierarchical network, which turns out to be the GrassLand food web according to H, H_{K}, H_{GRC}, H_{LF}, and the TRN-Yeast-1 network according to H_{PR}. The TRN-Yeast-1 network is the second most hierarchical in the sample according to H_{K}, H_{GRC} and H_{LF}, but receives only the 7th largest hierarchy value according to H.

Taken together, the comparison between the hierarchy measures revealed that our random walk hierarchy measure is consistent with the previously introduced hierarchy measures at large, but in the mean time it shows important differences when examined in details. This supports our view that grasping the signs of hierarchy in networks is a non-trivial task, and the listed hierarchy measures offer the characterisation of hierarchy from different as-

Type	Network	H	H_{K}	H_{GRC}	H_{LF}	H_{PR}
Electric	s5378 [5]	0.887	0.644	0.231	0.457	1.063
Citation	ArXiv-HepTh [6]	0.430	0.858	0.385	0.374	3.466
Food web	GrassLand [7]	1.551	1	0.961	1	2.157
Internet	p2p-1 [10]	0.524	0.753	0.598	0.531	1.895
Metabolic	E. coli [11]	0.447	0.131	0.043	0.031	2.181
Organization	Enron [13]	0.126	0.087	0.038	0.010	0.677
Regulatory	TRN-Yeast-1 [15]	0.262	0.995	0.934	0.984	8.601
Trust	WikiVote [20]	0.201	0.924	0.494	0.619	1.660
Language	English [21]	0.404	0.260	0.124	0.027	5.582
WWW	Google web [22]	0.258	0.356	0.216	0.040	2.605

Table S4: Comparing the different hierarchy measures on real networks. The network type is given in the 1st column, the references to the data sources are listed in the 2nd column. The random walk hierarchy H, defined in Eq.(11) in the main paper is given in the 3rd column, followed by Krackhars's hierarchy defined in Eq.(S1) in the 4th column, the Global Reaching Centrality given in Eq.(S2) in the 5th column, the link flow hierarchy defined in Eq.(S3) in the 6th column and the hierarchy based on the inhomogeneity of the PageRank given in Eq.(S16) listed in the 7th column.

pects. Our random walk hierarchy provides an important new alternative, measuring the extent of hierarchy in the network structure from a new point of view.

S8 The robustness of H

Finally, in this section we examine the robustness of H against random redirection of links in real networks. It is widely known that the data behind real networks can be noisy or even spurious in some cases, thus, a part of the links we take into account during the network analysis might not exist or point to a different node in reality. Relating to that, an important question to ask is how sensitive is our hierarchy measure to small changes in the network structure that mimic corrections due to spurious connections?

In Fig.S8. we show $\langle H\rangle$ obtained when the direction of links chosen at random was inverted as function of the fraction of modified links f for the networks listed in Table.S4. According to the results, H is quite robust against these changes in most of the systems. The largest deviation from the original H is observed in case of the Grassland food web, (shown by yellow diamonds in Fig.S8.), where $\langle H\rangle$ is showing a decaying tendency as a function of f. However, when inverting 1% of the links, the induced change

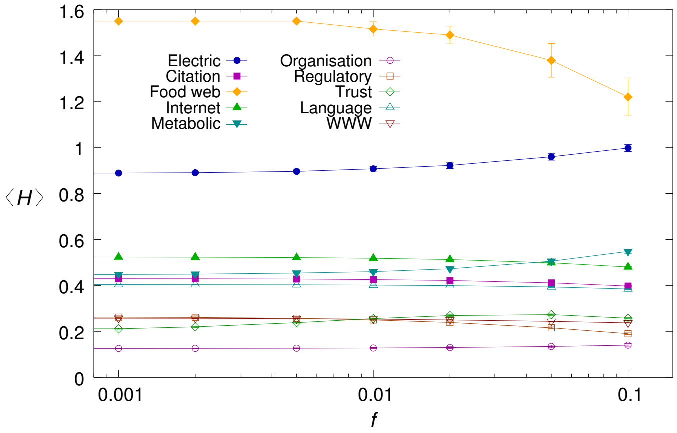

Figure S8: Robustness of H against random inversion of the link directions. The average random walk hierarchy, $\langle H\rangle$, is plotted as a function of the fraction of inverted links, f, for the networks listed in Table.S4. The colour and shape of the symbols encode the different systems, and the error-bars correspond to the standard deviation of the hierarchy measure, $\sigma(H)$. (In most cases the size of the symbols is larger than $\sigma(H)$).

in $\langle H\rangle$ is corresponding to only about 2.2% of the original H value, and if the fraction of inverted links is increased to 10%, the relative change in $\langle H\rangle$ compared to the original network is about 21.3%. The other systems involved in this study are even less sensitive to these random changes, with almost constant $\langle H\rangle$ curves in some of the cases.

I Fig.S9. we show the results for $\langle H\rangle$ when instead of inverting the direction of the randomly chosen links we simply rewire them to a randomly selected target node. The behaviour of $\langle H\rangle$ is very similar to what we have seen in Fig.S8: For most of the networks the random walk hierarchy shows a considerable robustness, as the value of $\langle H\rangle$ is very close to the original H even when 10% of the links is rewired. In this case the perturbation of the network has the largest effect on H for the Electric network s5378. However, even in this case the random rewiring of 1% of the links results in only about a 2.8% increase in $\langle H\rangle$, whereas if the fraction of rewired links is increased to 10%, the induced relative change in $\langle H\rangle$ compared to the original network is corresponding to about 18.5%.

Based on the above, H seems notably robust against random redirection of a small fraction of the links. This is relevant from the point of view of our analysis regarding the z-score of H in the main paper and in Sect.S5.

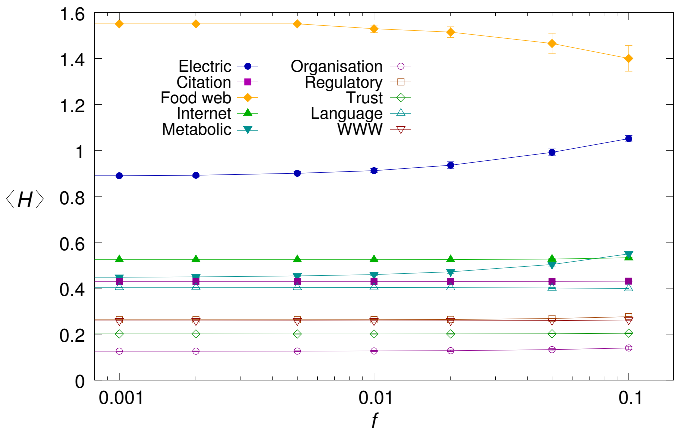

Figure S9: Robustness of H against random rewiring of the links. The average random walk hierarchy, $\langle H \rangle$ is plotted as a function of the fraction of rewired links, f, for the networks listed in Table.S4. The colour and shape of the symbols encode the different systems, and the error-bars correspond to the standard deviation of the hierarchy measure, $\sigma(H)$. (In most cases the size of the symbols is larger than $\sigma(H)$).

According to the results shown in Figs.S8-S9., the presence of spurious or noisy links can have only a very slight effect on the random walk hierarchy, (as long as these connections are rare). Therefore, the extremely high z-score values observed in some of the systems, (such as for example the citation networks), cannot be accounted for a small number of supposed spurious connections.

References

[1] Krackhardt, D. Graph theoretical dimensions of informal organizations. In Carley, K. & Prietula, M. (eds.) *Computational organization theory*, 89–111 (Lawrence Erlbaum Associates, Hillsdale, NJ, 1994).

[2] Mones, E., Vicsek, L. & Vicsek, T. Hierarchy measure for complex networks. *PLoS ONE* **7**, e33799 (2012).

[3] Luo, J. & Magee, C. L. Detecting evolving patterns of self-organizing networks by flow hierarchy measurement. *Complexity* **16**, 53–61 (2011).

[4] Corominas-Murtra, B., Goñi, J., Solé, R. V. & Rodrguez-Caso, C. On the origins of hierarchy in complex networks. *Proc. Natl. Acad. Sci. USA* **110**, 13316–13321 (2013).

[5] `http://courses.engr.illinois.edu/ece543/iscas89.html` (Date of access: 24/02/2012).

[6] Leskovec, J., Kleinberg, J. & Faloutsos, C. Graphs over time: densification laws, shrinking diameters and possible explanations. In *Proceedings of the eleventh ACM SIGKDD international conference on Knowledge discovery in data mining*, 177–187 (ACM, 2005).

[7] Dunne, J. A., Williams, R. J. & Martinez, N. D. Food-web structure and network theory: the role of connectance and size. *Proceedings of the National Academy of Sciences* **99**, 12917–12922 (2002).

[8] Martinez, N. D. Artifacts or attributes? Effects of resolution on the little rock lake food web. *Ecological Monographs* 367–392 (1991).

[9] Christian, R. R. & Luczkovich, J. J. Organizing and understanding a winter's seagrass foodweb network through effective trophic levels. *Ecological Modelling* **117**, 99–124 (1999).

[10] Leskovec, J., Kleinberg, J. & Faloutsos, C. Graph evolution: Densification and shrinking diameters. *ACM Transactions on Knowledge Discovery from Data (TKDD)* **1**, 2 (2007).

[11] Jeong, H., Tombor, B., Albert, R., Oltvai, Z. N. & Barabási, A.-L. The large-scale organization of metabolic networks. *Nature* **407**, 651–654 (2000).

[12] Cross, R., Parker, A., Christensen, C. M., Anthony, S. D. & Roth, E. A. *The hidden power of social networks* (Audio-Tech Business Book Summaries, Incorporated, 2004).

[13] Klimt, B. & Yang, Y. Introducing the enron corpus. In *CEAS* (2004).

[14] Freeman, S. & Freeman, L. *Social science research reports 46* (University of California, Irvine, CA, 1979).

[15] Balaji, S., Babu, M. M., Iyer, L. M., Luscombe, N. M. & Aravind, L. Comprehensive analysis of combinatorial regulation using the transcriptional regulatory network of yeast. *Journal of molecular biology* **360**, 213–227 (2006).

[16] Milo, R. *et al.* Network motifs: simple building blocks of complex networks. *Science* **298**, 824–827 (2002).

[17] Van Duijn, M. A., Zeggelink, E. P., Huisman, M., Stokman, F. N. & Wasseur, F. W. Evolution of sociology freshmen into a friendship network. *Journal of Mathematical Sociology* **27**, 153–191 (2003).

[18] Richardson, M., Agrawal, R. & Domingos, P. Trust management for the semantic web. In *The Semantic Web-ISWC 2003*, 351–368 (Springer, 2003).

[19] Milo, R. *et al.* Superfamilies of evolved and designed networks. *Science* **303**, 1538–1542 (2004).

[20] Leskovec, J., Huttenlocher, D. & Kleinberg, J. Signed networks in social media. In *Proceedings of the SIGCHI Conference on Human Factors in Computing Systems*, 1361–1370 (ACM, 2010).

[21] i Cancho, R. F. & Solé, R. V. The small world of human language. *Proceedings of the Royal Society of London. Series B: Biological Sciences* **268**, 2261–2265 (2001).

[22] Palla, G., Farkas, I. J., Pollner, P., Derenyi, I. & Vicsek, T. Directed network modules. *New Journal of Physics* **9**, 186 (2007).

[23] Albert, R., Jeong, H. & Barabási, A.-L. Internet: Diameter of the world-wide web. *Nature* **401**, 130–131 (1999).

[24] Adamic, L. A. & Glance, N. The political blogosphere and the 2004 U.S. election: divided they blog. In *Proceedings of the 3rd International Workshop on Link discovery*, 36–43 (ACM, 2005).

[25] Brin, S. & Page, L. The anatomy of a large-scale hypertextual Web search engine. *Computer Networks and ISDN Systems* **33**, 107–117 (1998).
